# Energy-Conserving Enzyme Systems Active During Syntrophic Acetate Oxidation in the Thermophilic Bacterium *Thermacetogenium phaeum*

**DOI:** 10.3389/fmicb.2019.02785

**Published:** 2019-11-29

**Authors:** Anja Keller, Bernhard Schink, Nicolai Müller

**Affiliations:** ^1^Department of Biology, Universität Konstanz, Konstanz, Germany; ^2^Konstanz Research School Chemical Biology, Konstanz, Germany

**Keywords:** syntrophic acetate oxidation, acetogenesis, methylene-THF reductase, membrane–bound formate dehydrogenase, Wood-Ljungdahl pathway

## Abstract

The thermophilic acetogen *Thermacetogenium phaeum* uses the Wood-Ljungdahl pathway (WLP) in both directions, either for the production of acetate from various compounds or for the oxidation of acetate in syntrophic cooperation with methanogens. In this study, energy–conserving enzyme systems in *T. phaeum* were investigated in both metabolic directions. A gene cluster containing a membrane-bound periplasmically oriented formate dehydrogenase directly adjacent to putative menaquinone synthesis genes was identified in the genome. The protein products of these genes were identified by total proteome analysis, and menaquinone MK-7 had been found earlier as the dominant quinone in the membrane. Enzyme assays with membrane preparations and anthraquinone-2,6-disulfonate as electron acceptor verified the presence of a quinone-dependent formate dehydrogenase. A quinone-dependent methylene–THF reductase is active in the soluble fraction and in the membrane fraction. From these results we conclude a reversed electron transport system from methyl-THF oxidation to CO_2_ reduction yielding formate as reduced product which is transferred to the methanogenic partner. The redox potential difference between methyl-THF (E_o_’ = −200 mV) and formate (E_o_’ = −432 mV) does not allow electron transfer through syntrophic formate removal alone. We postulate that part of the ATP conserved by substrate-level phosphorylation has to be invested into the generation of a transmembrane proton gradient by ATPase. This proton gradient could drive the endergonic oxidation of methyl-THF in an enzyme reaction similar to the membrane-bound reversed electron transport system previously observed in the syntrophically butyrate-oxidizing bacterium *Syntrophomonas wolfei*. To balance the overall ATP budget in acetate oxidation, we postulate that acetate is activated through an ATP-independent path via aldehyde:ferredoxin oxidoreductase (AOR) and subsequent oxidation of acetaldehyde to acetyl-CoA.

## Introduction

The Wood-Ljungdahl pathway (WLP) or reductive acetyl-CoA pathway is the central pathway in acetogens and most strictly anaerobic acetate-oxidizing bacteria (AOB). Although the WLP was investigated in depth the mechanism of energy conservation of most acetogens and AOB remained unclear since no net ATP is gained in this pathway by substrate level phosphorylation (Schuchmann and Müller, 2014). For *Acetobacterium woodii*, the mechanism of energy conservation was elucidated completely (Biegel et al., 2009, 2011; Biegel and Müller, 2010; Hess et al., 2013; Bertsch et al., 2015) and also the one in *Moorella thermoacetica* was studied in detail (Huang et al., 2012; Wang et al., 2013; Mock et al., 2014). *A. woodii* conserves energy with the help of a *Rhodobacter* nitrogen fixation (Rnf) complex which pumps sodium ions across the membrane while reduced ferredoxin (Fd^2–^) is oxidized with NAD^+^ in an exergonic reaction (Biegel and Müller, 2010). Other modes of energy conservation were hypothesized before. The thermophile *M. thermoacetica* was shown to have a heterohexameric methylene-THF reductase (MTHFR) (Mock et al., 2014) which does not catalyze the reduction of methylene-THF with NADH. The genes for this enzyme are located in a cluster containing genes annotated as a heterodisulfide reductase (Hdr) enzyme complex. In the same study, it was proposed that the MTHFR could be coupled via formate dehydrogenase to the Ech hydrogenase, similar to a membrane-bound formate hydrogenlyase complex found in *Escherichia coli*. This system could be used to create a proton gradient across the membrane and thus conserve energy during acetogenesis (Mock et al., 2014). In contrast, *A. woodii* lacks this putatively proton translocating system and instead has a heterotrimeric NADH-oxidizing methylene-tetrahydrofolate (THF) reductase which is not coupled to energy conservation (Bertsch et al., 2015).

Only little is known so far about the biochemistry of syntrophic acetate-oxidizing bacteria (SAOB). SAOB are hard to isolate and to cultivate. To date only six defined cultures are known (Schnürer et al., 1996; Hattori et al., 2000; Balk et al., 2002; Westerholm et al., 2010, 2011; Timmers et al., 2018). These cultures do not reach high cell densities, and investigations in cell-free systems are challenged with the problem that the SAOB have to be separated from their methanogenic partners to obtain cell suspensions containing only the bacterial component. One of the strains whose physiology was investigated in more detail is *Clostridium ultunense*, a mesophilic bacterium that oxidizes acetate in a triculture with a hydrogen- and formate-utilizing methanogen MAB1 and a further bacterium, strain TRX1 (Schnürer et al., 1996). The difficulties of mass cultivation for enzyme assays of syntrophic acetate oxidizers can be overcome by using proteomic and genomic approaches. A recent study compared the genomes of all defined SAOB co-cultures that have been sequenced so far (Manzoor et al., 2018).

For the present study, *Thermacetogenium phaeum* was chosen as a model organism for SAOB as its genome sequence is available. It poses a special case of SAOB due to its thermophilic lifestyle, with a temperature range between 40 and 65°C and an optimum growth temperature of 58°C, that facilitates acetate conversion to CO_2_ and CH_4_ (Hattori et al., 2000; Oehler et al., 2012). *T. phaeum* is able to revert the WLP and thus oxidizes acetate in syntrophic cooperation with *Methanothermobacter thermautotrophicus* strain TM but uses as well hydrogen plus CO_2_ to form acetate in axenic cultures (Hattori et al., 2000, 2005). Recently, growth of *T. phaeum* with acetate, ethanolamine, methanol, and ethanol was characterized by proteomic analysis and enzyme assays (Keller et al., 2019). In the current study, the focus will be on syntrophic growth with acetate, and on axenic growth with formate or hydrogen plus CO_2_. Enzyme systems that are possibly involved in energy conservation such as membrane-bound formate dehydrogenases or hydrogenases as well as the MTHFR are studied in detail.

## Materials and Methods

### Origin of Organisms and Culture Conditions

Axenic cultures of *T. phaeum* strain PB (DSM 26808) as well as the syntrophic co-culture with *M. thermautotrophicus* strain TM were obtained from the German Culture Collection (DSMZ, Braunschweig, Germany). Cultures were grown anaerobically in modified freshwater medium DSM880 as described before (Keller et al., 2019) at 55°C in the dark without shaking. The axenic culture of *T. phaeum* was grown with formate or hydrogen/CO_2_, whereas the syntrophic co-culture was grown with acetate as substrate. Cultivation with hydrogen/CO_2_ (79%/21% v/v) was performed by flushing the headspace (70 ml) of 150 ml bottles for 1 min at an overpressure of 1 bar. Formate and acetate were autoclaved in 3 M stock solutions and then added to the cultures to 40 mM final concentration. Cultures were transferred at least 10 times [corresponding to approximately 22 (H_2_/CO_2_) or 30 (formate) cell generations] with the respective substrates to assure complete adaptation before growth curves were recorded and proteome analysis was performed. For quantification of growth, four bottles were filled each with 45 ml medium and 5 ml pre-culture. Increase in optical density was monitored with a Jenway 6300 spectrophotometer (Staffordshire, United Kingdom) at 600 nm. Substrate depletion and product formation was monitored by HPLC with a Shimadzu system as described before (Keller et al., 2019). Compounds were separated at 60°C on a Rezex^TM^ RHM-Monosaccharide H^+^ (8%) ion exchange resin column (LC column 300 × 7.8 mm, 00H-0132-K0, Phenomenex, Los Angeles, CA, United States).

### Preparation of Cell-Free Extract and Subcellular Fractions

The preparation of cell-free extracts and subcellular fractions for enzyme activity measurements was carried out under strictly anoxic conditions in an anoxic glove box (Coy, Ann Arbor, MI, United States). Centrifugation was performed in air-tight vessels, and buffers were made anoxic by alternately applying vacuum and 100% N_2_ three times under vigorous stirring. Cultures were harvested by centrifugation at 7,000 × *g* for 15 min at 4°C and washed once with 50 mM Tris–HCl buffer, pH 7.5, containing 3 mM dithiothreitol (DTT). The co-culture was separated by a self-assembling Percoll gradient (70% Percoll in distilled water containing 250 mM sucrose) adapted from Luo et al. (2002) and Enoki et al. (2011) as described before (Keller et al., 2019). The gradient tubes were centrifuged for 1 h at 4°C at 45,000 × *g* in a type 70-Ti rotor in an Optima LE-80K ultracentrifuge (Beckman Coulter, Brea, CA, United States). Cells of *T. phaeum* were enriched in the upper one of the two bands and the cells were collected and washed with 50 mM Tris–HCl, pH 7.5, containing 3 mM DTT. Percoll-separated *T. phaeum* cells of syntrophic cultures or *T. phaeum* cells of axenic cultures were suspended in 3 ml Tris–HCl buffer, pH 7.5, containing 3 mM DTT, and disrupted by at least three passages through a French pressure cell (Aminco, Silver Spring, MD, United States) operated at 137 MPa. The crude extract was centrifuged at room temperature at 11,300 × *g* for 5 min to clear it from cell debris and unopened cells. The soluble fraction containing cytoplasmic and periplasmic enzymes was obtained by ultracentrifugation at 100,000 × *g* in an Optima TL-ultracentrifuge using a TLA110-rotor (Beckman Coulter, Brea, CA, United States) for 1 h. The pellet was washed once with 50 mM Tris–HCl, pH 7.5, containing 3 mM DTT, and after the second centrifugation the pellet was suspended in 0.8 ml and defined as membrane fraction. The soluble fraction was further separated via an anion exchange column (Q-sepharose, HiTrapQ HP column, 5 ml, GE Healthcare, Pittsburgh, PA, United States) manually operated with syringes as described by Keller et al. (2019). First, 0.8 ml of the soluble fraction was applied and the column was washed with five column volumes of 50 mM Tris–HCl, pH 7.5, containing 3 mM DTT. Fraction 1 was eluted with two column volumes of Tris–HCl buffer containing additional 200 mM NaCl and then fraction 2 was eluted with Tris–HCl buffer containing 1 M NaCl.

### Mass Spectrometry

Mass spectrometry was performed at the Proteomics facility of the University of Konstanz as described before (Keller et al., 2019). The membrane fraction was cleared from interfering lipids by suspending the membrane pellet in 10% SDS. The solubilized membrane pellet was mixed with loading dye (0.125 M Tris–HCl, pH 6.8, 2% (w/v) SDS, 25% glycerol, 0.01% (w/v) bromophenolblue and 5% β-mercaptoethanol) at a ratio of 1:1, heated to 98°C for 10 min, and was run about 2 cm into a 12% SDS gel (Laemmli, 1970). The gel was stained with colloidal Coomassie (Neuhoff et al., 1988; Schmidt et al., 2013) and the band containing the protein was excised. Samples were digested by trypsin treatment and analyzed by liquid chromatography nanospray tandem mass spectrometry (LC-MS/MS) using an Eksigent nano–HPLC and an LTQ–Orbitrap mass spectrometer (Thermo Fisher, Waltham, MA, United States) as described before (Keller et al., 2019). The ion chromatogram was analyzed using the Proteome Discoverer software (Thermo Fisher, Waltham, MA, United States) and the areas of the respective peaks were integrated for semi-quantitative analysis of relative protein abundances.

### Enzyme Activity Measurements

All enzyme activity measurements were performed anoxically in glass cuvettes sealed with rubber stoppers which were flushed with 100% N_2_. Activity measurements were carried out at least in triplicate in a Jasco V630 or V730 spectrophotometer (Tokyo, Japan) at 55°C with 50 mM Tris–HCl buffer, pH 7.5, containing 3 mM DTT if not stated otherwise.

#### Formate Dehydrogenase

Formate dehydrogenase was assayed according to Schmidt et al. (2014) and Keller et al. (2019). Electron acceptors used were either 0.5 mM anthraquinone-2,6-disulfonate (AQDS) [ε_408_ = 7.2 mM^–1^ cm^–1^ (Liu et al., 2007; Shi et al., 2012)], 1 mM benzyl viologen (BV) [BV: ε_578_ = 8.65 mM^–1^cm^–1^ (McKellar and Sprott, 1979)], 0.25 mM NAD^+^ [ε_340_ = 6.3 mM^–1^ cm^–1^ (Ziegenhorn et al., 1976)] or 16 μM oxidized ferredoxin (Fd_ox_) [ε_390_ = 30 mM^–1^ cm^–1^ (Gersonde et al., 1971)]. Reduction of AQDS was monitored at 408 nm, of BV at 578 nm, of NAD^+^ at 340 nm and of Fd_ox_ at 390 nm. Reactions were started by addition of 5 mM sodium formate.

#### Hydrogenases

Hydrogenases were measured analogous to formate dehydrogenase with the electron acceptors 0.5 mM AQDS, 1 mM BV, 0.25 mM NAD^+^ and 16 μM Fd_ox_. The reaction was started by injection of 100 μl hydrogen into the head space according to Keller et al. (2019).

#### NADH:Acceptor Oxidoreductase

NADH:acceptor oxidoreductase was measured with 0.5 mM NADH and 0.5 mM AQDS. To monitor the reaction, reduction of AQDS was followed at 408 nm. Formate dehydrogenase, hydrogenase and NADH:acceptor oxidoreductase activity were measured in soluble and membrane fractions of *T. phaeum* cells grown in syntrophic co-culture with acetate.

#### Methylene-THF Reductase (MTHFR)

Methylene-THF reductase was measured with 0.25 mM NADH or 0.25 mM NADPH as electron donor and methylene-THF as electron acceptor which was synthesized directly in the buffer as described in detail in Bertsch et al. (2015). For this purpose, 1.5 mM formaldehyde and 0.5 mM THF were mixed in 50 mM Tris buffer, pH 7.0, containing 3 mM DTT. Controls with formaldehyde alone were performed to rule out side reactions such as methanol dehydrogenase. To examine an electron bifurcation function of the MTHFR 16 μM Fd_ox_ was added to 0.25 mM NADH and oxidation of NADH was monitored. Oxidation of NADH and NADPH was followed at 365 nm [ε_365_ = 3.441 mM^–1^ cm^–1^ (Ziegenhorn et al., 1976)]. Furthermore, the MTHFR was assayed with 0.2 mM methyl-THF and 0.5 mM NAD^+^, 1 mM BV and 0.5 mM AQDS as electron acceptors modified after (Rosner and Schink, 1995; Bertsch et al., 2015). The enzyme was assayed in 50 mM Tris buffer, pH 7.5, containing 3 mM DTT, and the reaction was started by addition of methyl-THF. NAD^+^ reduction was monitored at 340 nm [ε_340_ = 6.3 mM^–1^ cm^–1^ (Ziegenhorn et al., 1976)], BV reduction at 578 nm [BV: ε_578_ = 8.65 mM^–1^cm^–1^ (McKellar and Sprott, 1979)], and AQDS reduction at 408 nm [ε_408_ = 7.2 mM^–1^ cm^–1^ (Liu et al., 2007; Shi et al., 2012)].

#### Methylene-THF Dehydrogenase (MTHFD)

Methylene-THF dehydrogenase was assayed with 0.25 mM NAD^+^ and 0.25 mM NADP^+^ as electron acceptors and methylene-THF as electron donor, which was synthesized as described above. The reduction of NAD^+^ and NADP^+^ was monitored at 365 nm. Activities of MTHFR and MTHFD were assayed in the soluble fraction and its subfractions 1 and 2, as well as in the membrane fractions of acetate-grown cells.

### Comparison of Gene Clusters

The methylene-THF encoding gene clusters of *A.* woodii WB1 (DSM 1030), *M. thermoacetica* (ATCC 39073) and *T. phaeum* PB (DSM12270) as well as the gene cluster containing the periplasmically oriented formate dehydrogenase of *T. phaeum* and *Syntrophomonas wolfei* Goettingen (DSM2245B) were compared with the help of the IMG genome BLAST tool^1^ using the blastp program comparing amino acid sequences. Transmembrane domains were predicted with TMHMM (v.2.0, URL)^2^, and signal peptides were predicted with SignalP 5.0^3^. Selenocysteine insertion motifs were identified using the bSECISearch tool (Zhang and Gladyshev, 2005)^4^.

## Results

### Growth With Formate or Hydrogen/CO_2_

**FIGURE 1:**
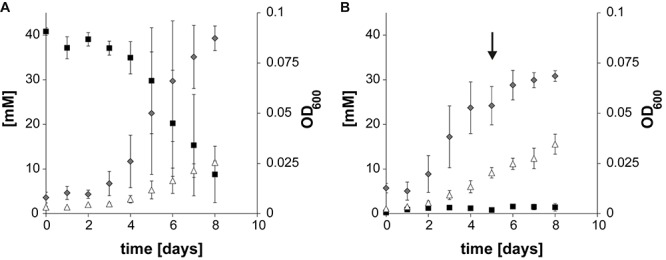
Growth curves of *Thermacetogenium phaeum* depicting substrate depletion, product formation, and OD_600_ increase. **(A)** Axenic growth with formate. **(B)** Axenic growth with hydrogen/CO_2_. The arrow marks the time point when the culture was refed with H_2_/CO_2_. Gray diamonds depict OD_600_, black squares depict formate concentration and white triangles depict acetate. All concentrations are given in mM ± standard deviation.

Axenic cultures of *T. phaeum* were grown with 40 mM formate or hydrogen/CO_2_, respectively. For growth with hydrogen/CO_2_ (79%/21%) the headspace of the bottles was flushed for 1 min. As described earlier (Keller et al., 2019), syntrophic cultures of *T. phaeum* with *M. thermautotrophicus* grown with 40 mM acetate needed 21 days to reach early stationary phase with a doubling time of 42 h as described before (Keller et al., 2019). Cultures grown with formate needed 8 days and with hydrogen/CO_2_ 5 days to reach stationary phase, with doubling times of 25 to 30 h during exponential growth phases. Growth was very poor and the average change in OD_600_ was 0.042 for hydrogen/CO_2_ which, however, could be increased by flushing the headspace again with hydrogen/CO_2_. Cultures grown with formate reached an average OD_600_ of 0.07 (Figure 1).

### Total Proteome Analysis

**FIGURE 2:**
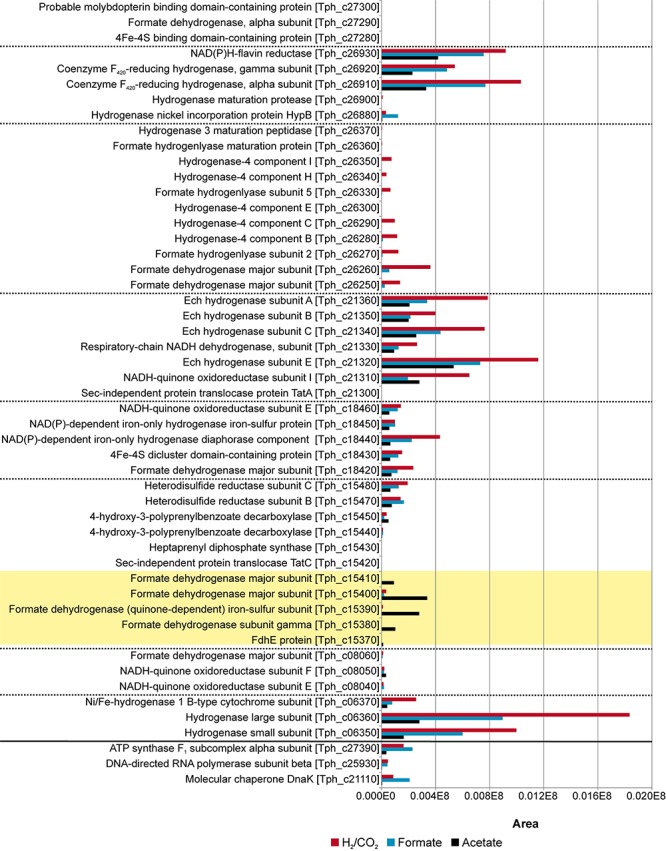
Proteome data of all formate hydrogenases and hydrogenases obtained during growth of *T. phaeum* with formate, hydrogen/CO_2_, and acetate. All expressed genes of the different gene clusters are shown in the graph. The clusters are separated by dashed lines. Proteins of the membrane bound, periplasmically oriented formate dehydrogenase that are dominantly present during syntrophic growth with acetate are highlighted in yellow. Housekeeping proteins are depicted beneath the solid line. The proteome data for cultivation with acetate is taken from Keller et al. (2019). The relative abundance of the respective proteins was semi-quantitatively analyzed using the area values of the corresponding peaks of the ion chromatogram and using the Proteome Explorer software (Thermo Fisher). Shown are non-normalized area values in relation to area values of housekeeping proteins (ATPase, RNA-polymerase and molecular chaperone DnaK).

**FIGURE 3:**
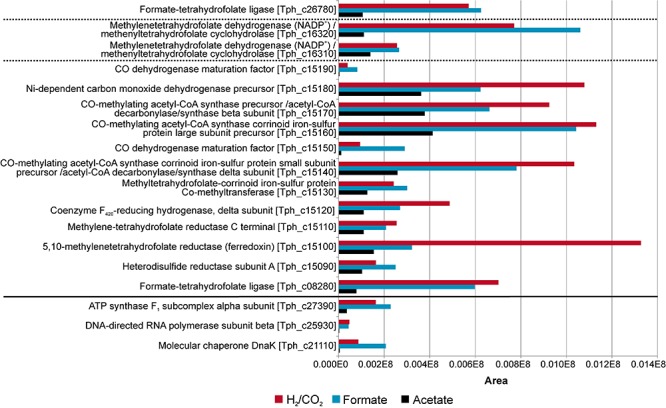
Proteome data of all enzymes of the Wood-Ljungdahl pathway (WLP) obtained during growth of *T. phaeum* with formate, hydrogen/CO_2_, and acetate. The clusters are separated by dashed lines. Housekeeping proteins are depicted beneath the solid line. The proteome data for cultivation with acetate is taken from Keller et al. (2019). The relative abundance of the respective proteins was semi-quantitatively analyzed using the area values of the corresponding peaks of the ion chromatogram and using the Proteome Explorer software (Thermo Fisher). Shown are non-normalized area values in relation to area values of housekeeping proteins (ATPase, RNA-polymerase and molecular chaperone DnaK).

Total proteome analysis was done with both the soluble fraction and the membrane fraction after syntrophic growth with acetate and growth with formate or hydrogen/CO_2_ (Supplementary Table S1). All four hydrogenase systems and one formate hydrogenlyase system (FHL) encoded in the genome (Oehler et al., 2012) were identified in the proteome at different levels of abundance (Figure 2). Non-F_420_–reducing hydrogenase (gene locus tags Tph_c26910-26930), membrane-bound Ech hydrogenase (Tph_c21310-21360), NAD(P)-dependent iron-only hydrogenase (Tph_c18430- 18460) and a periplasmic [NiFeSe] hydrogenase (Tph_c06350- 06370) were identified in the proteome during growth with acetate, formate, and hydrogen/CO_2_ (Figure 2). A FHL was present during growth with hydrogen/CO_2_. This FHL system comprises 9 subunits (Tph_c26250- c26370). These subunits are two formate dehydrogenase subunits (Tph_c26250- c_26260), two FHL subunits (Tph_c26270, Tph_c26330) and five hydrogenase-4 (FHL) subunits (Tph_c26280- c26300, Tph_c26340- c26350). Besides the formate dehydrogenase genes present in the FHL cluster, there are five more formate dehydrogenase genes encoded in the genome of *T. phaeum*. The formate dehydrogenase (Tph_18420) whose gene is located next to the one of an NAD(P)-dependent iron-only hydrogenase was present in the proteome during growth with all three substrates. Two formate dehydrogenase gene clusters (Tph_c21680- 21660, Tph_c08060- 08040) were found to be located next to genes annotated as a putative NADH:quinone oxidoreductase. One of the latter formate dehydrogenase gene clusters (Tph_c21680- 21660) was apparently not expressed under the applied growth conditions as the respective proteins were not identified in the proteome. The gene cluster of the other formate dehydrogenase (Tph_c08060-08040) was only partially expressed during growth with acetate but constitutively expressed during growth with formate and hydrogen/CO_2_ as judged from the presence of the respective proteins. Another formate dehydrogenase (Tph_c27290) was present only during growth with acetate at a very low level, however, in an earlier study, this protein was found to be moderately abundant during syntrophic growth with ethanol or ethanolamine (Keller et al., 2019). Membrane-bound formate dehydrogenase (Tph_c15380- 15410) was identified in the proteome during growth with acetate and not during growth with formate or hydrogen. Enzymes of the WLP were present in the proteome during growth with formate or hydrogen/CO_2_ (Figure 3). The presence of all enzymes of the WLP during growth with acetate was shown before (Keller et al., 2019).

### Analysis of the Methylene-THF Reductase Gene Cluster

**FIGURE 4:**
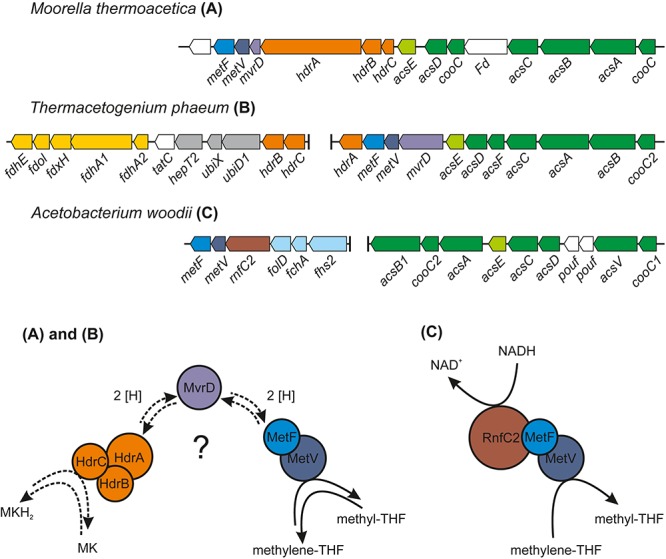
Comparison of the organization of the gene cluster containing the CODH/ACS–encoding genes and the genes for MTHFR of *T. phaeum* with the corresponding gene clusters of *M. thermoacetica* and *Acetobacterium woodii*. The proposed system for the electron transfer of the MTHFR in *T. phaeum* and *M. thermoacetica* is depicted in **(A)** and **(B)** and the protein complex responsible for methylene-THF reduction in *A. woodii* is depicted in **(C)** [adapted from Bertsch et al. (2015)]. MK, oxidized menaquinone; MKH_2_, menaquinol; MvrD, methyl-viologen-reducing hydrogenase subunit D; the native electron carrier is still unknown and MvrD is one potential candidate (labeled with a question mark). Activities of MTHFR described in this study were measured with artificial electron acceptors.

The genes for the enzymes of the WLP were found to be clustered in two different locations in the genome of *T. phaeum*. The first cluster contains genes for MTHFD and cyclohydrolase (Tph_c16310- Tph_16320). The amino acid sequence of the two subunits of the enzyme combining MTHFD and cyclohydrolase activity exhibit 50% (Tph_c16320) and 54% identity (Tph_c16310) compared to the ones of *M. thermoacetica*. Identities with the homologs in *A. woodii* are substantially lower, with 35% and 29%, respectively. The second cluster contains the genes encoding CODH/ACS (structural precursor genes and their maturation factors; Tph_c15140- Tph_c15190), methyl-tetrahydrofolate-corrinoid iron-sulfur protein Co-methyltransferase (Tph_c15130) and MTHFR (Tph_c15100- Tph_c15110). MTHFR subunits of *T. phaeum* show high similarity to the MetF (Tph_c15100, 39% identity) and MetV (Tph_c15110 34% identity) of *A. woodii* (Table 1; Keller et al., 2019). When comparing the gene clusters of *A. woodii* and *M. thermoacetica*, differences in the composition of this gene cluster can be observed (Figure 4). Compared to *A. woodii*, *T. phaeum* lacks the gene that is annotated as *rnfC2* and its product was postulated as the NADH-binding subunit of the MTHFR in *A. woodii* (Bertsch et al., 2015; Keller et al., 2019). Analogous to *M. thermoacetica*, in *T. phaeum*, an *hdrA* gene is located in the gene cluster directly adjacent to the MTHFR. The amino acid sequences of HdrA are identical to 31%. Furthermore, there is a coenzyme F_420_-reducing hydrogenase subunit (Tph_c15120) that shows 40% identity to the one of *M. thermoacetica*. These two enzymes are not present in *A. woodii*. The genes for the subunits HdrB and HdrC present in *M. thermoacetica* are not located in the methylene-THF containing gene cluster of *T. phaeum*. However, there is a gene coding for an HdrB (Tph_c15470) subunit whose amino acid sequence has an identity of 42% and a gene for an HdrC (Tph_c15480) subunit whose amino acid sequence has an identity of 36% to the one of *M. thermoacetica* in a different gene cluster next to a formate dehydrogenase gene and to the quinone synthesis genes.

**TABLE 1:** Comparison of genes of the cluster coding for CODH and MTHFR of *Thermacetogenium phaeum* to genes of *Moorella thermoacetica* and *Acetobacterium woodii*.

**Gene name**	***T. phaeum***	***M. thermoacetica***		***A. woodii***	
			
	**Locus tag Tph_c**	**Locus tag Moth_**	**Identity [%]**	**Locus tag Awo_c**	**Identity [%]**
Hypothetical protein	15080	No identity		No identity	
Heterodisulfide reductase subunit A	15090	1194	31	No identity	
5,10-methylene-tetrahydrofolate reductase (ferredoxin)	15100	1191	66	09310	39
Methylene-tetrahydrofolate reductase C terminal	15110	1192	55	09290 09300	38 34
Coenzyme F_420_-reducing hydrogenase, delta subunit	15120	1193	40	10560	31
Methyl-tetrahydrofolate-corrinoid iron-sulfur protein Co-methyltransferase	15130	1197	62	10730	39
CO-methylating acetyl-CoA synthase corrinoid iron-sulfur protein small subunit precursor/acetyl-CoA decarbonylase/synthase delta subunit	15140	1198	57	10710	37
CO dehydrogenase maturation factor	15150	1199	57	10670 10750	46 32
CO-methylating acetyl-CoA synthase corrinoid iron-sulfur protein large subunit precursor	15160	1201	58	10720	42
CO-methylating acetyl-CoA synthase precursor/acetyl-CoA decarbonylase/synthase beta subunit	15170	1202	59	10760	44
Ni-dependent carbon monoxide dehydrogenase precursor	15180	1203	57	10740	40
CO dehydrogenase maturation factor	15190	1204	56	10750	42

*Comparison was performed with the IMG genome BLAST tool.*

### Analysis of a Putatively Periplasmically Oriented Formate Dehydrogenase Gene Cluster

During growth with acetate, the genes coding for a membrane-bound formate dehydrogenase (Tph_c15370- c15410) were expressed. Genes coding for this enzyme system were found to be located next to quinone synthesis genes (Tph_c15430- c15460), to two genes of subunits of a heterodisulfide reductase (*hdrB* and *hdrC*, Tph_c15470- Tph_c15480) and to one gene of a subunit of a sec-independent TAT translocase (*tatC*, Tph_c15420) (Figure 4). Another gene for a subunit of the TAT translocase complex can be found elsewhere in the genome and is located next to the gene coding for Ech hydrogenase (*tatA*, Tph_c21300). The formate dehydrogenase complex consists of two genes coding for subunits containing *trans-*membrane helices; first a formate dehydrogenase gamma subunit gene (Tph_c15380) and second a quinone-dependent subunit gene (Tph_c15390). One of the remaining two subunit genes (*fdhA2*, Tph_c15410) carries a signal sequence for the Twin-arginine translocation pathway which is lacking in the other subunit (analyzed with SignalP 5.0 and automatic annotation in IMG). A selenocysteine insertion sequence (SECIS)-search of the nucleotide sequence of *fdhA2* (Tph_c15410) revealed that the proteins of this gene and the protein of the adjacent gene *fdhA1* coding for a large formate dehydrogenase subunit (Tph_c15400) are linked through selenocysteine incorporation, meaning that these two genes are translated into one single protein (Zhang and Gladyshev, 2005). In contrast to Oehler et al. (2012), we therefore suggest that this formate dehydrogenase complex is membrane bound, and that the fused protein of the genes *fdhA1* and *fdhA2* (Tph_c15400 and Tph_c15410) is located at the periplasmically oriented side of the enzyme complex. Consequently, the complete formate dehydrogenase complex consists of three protein subunits, namely two proteins with transmembrane helices (Tph_c15380 and fdh subunit gamma, Tph_c15390) and one large periplasmic subunit. The protein of the gene annotated as *fdhE* (Tph_c15370) is responsible for maturation of the formate dehydrogenase complex. Analysis of the gene neighborhood of the quinone-dependent formate dehydrogenase (Tph_c15390) with the same COG hit in IMG showed similarity with the respective gene neighborhood in *S. wolfei* and *Syntrophomonas zehnderi*. Yet, these strains do not have a heterodisulfide reductase encoded in the same gene cluster. In an IMG gene neighborhood search with this heterodisulfide reductase beta subunit, *Syntrophaceticus schinkii* shows the highest similarity. If the amino acid sequence of heterodisulfide reductase is searched against the genome of *S. wolfei* with the IMG BLAST tool, it shows 47% identity for the beta subunit and 43% for the HdrC subunit. The amino acid sequences of the formate dehydrogenase subunits FdhA1 (Tph_c15400, Swol_0799) showed 56% similarity and FdhA2 (Tph_c15410, Swol_0800) showed 53%, the iron-sulfur subunit (Tph_c15390, Swol_0798) had 54%, the gamma subunit (Tph_c15380, Swol_0797) 46% and the formate accessory protein (Tph_c15370, Swol_0796) had only 28% identity. In contrast to the iron-sulfur subunit (Tph_c15390), its homolog in *S. wolfei* (Swol_0796) does not have transmembrane helices. *M. thermoacetica* was shown to have a periplasmically oriented formate dehydrogenase (gene locus tags Moth_0450-0452) as well. The amino acid sequences of the major subunit (Moth_0450) showed 23 to 30% identity to the major subunits (Tph_c15400-15410) of the periplasmically oriented formate dehydrogenase of *T. phaeum*. The iron-sulfur complex containing subunit (Moth_0451) exhibits 30% identity with the quinone-dependent subunit (Tph_c15390) of *T. phaeum* and the gamma subunit (Moth_0452) shows 33% identity to the gamma subunit (Tph_c15380) of *T. phaeum*.

### Activities of Key Enzymes

#### Methylene-THF Reductase (MTHFR) and Methylene-THF Dehydrogenase (MTHFD)

Activities of MTHFR and MTHFD were assayed photometrically in the following subcellular fractions: membrane fraction, soluble fraction, and fraction 1 and 2 which were soluble fractions eluted from an anion exchange column with 200 mM NaCl or 1 M NaCl, respectively. MTHFR was measured with methylene-THF and NADH or NADPH as electron donors in the direction of methyl-THF formation. Activity with NADH was observed only in fraction 2 (Table 2), which is most likely due to the presence of MTHFD in the soluble fraction which immediately reduces the produced NAD^+^ through oxidation of methylene-THF. Therefore, both MTHFR and MTHFD have to be separated to properly assess their individual activity with methylene-THF. No activity was observed with NADPH. Addition of Fd_ox_ that was purified from *Clostridium pasteurianum* did not lead to increased activity. This test was run to check for a possible bifurcating enzyme reaction that could enable endergonic oxidation of methyl-THF with NAD^+^ by exergonic oxidation of reduced ferredoxin with another molecule of NAD^+^. MTHFR was measured also in the oxidative direction with methyl-THF and NAD^+^, benzyl viologen (BV) or anthraquinone-2,6-disulfonate (AQDS) as electron acceptor. No activity of the MTHFR was observed for methyl-THF oxidation with NAD^+^ in the soluble or membrane fraction. Instead, methyl-THF oxidizing enzyme activity was found with the artificial electron acceptors BV and AQDS and can therefore be considered as NAD^+^-independent. The highest activity with benzyl viologen was detected in the membrane fraction with 2598 mU/mg protein and the second highest one in fraction 2 with 477 mU/mg protein. Activities with the artificial quinone-analogous acceptor AQDS were generally lower and in the range of 4 mU/mg protein (membrane fraction) to 24 mU/mg protein (soluble fraction).

**TABLE 2:** Activities of methylene-THF reductase and methylene-THF dehydrogenase measured with various electron acceptors in different subcellular fractions of cells grown syntrophically with acetate.

**Substrate**	**Electron carrier**	**Activity [mU/mg protein]**			
		
		**SF**	**Fraction 1**	**Fraction 2**	**MF**
**Methylene-THF reductase**					
Methylene-THF	NADH	bd^a^	bd^a^	169 ± 9	bd^a^
Formaldehyde	NADH	5 ± 0	bd^a^	bd^a^	bd^a^
Methylene-THF	NADPH	−^b^	−^b^	bd^a^	−^b^
Formaldehyde	NADPH	−^b^	−^b^	bd^a^	−^b^
Methyl-THF	NAD^+^	bd	−^b^	−^b^	bd
Methyl-THF	BV	16 ± 9	bd^a^	477 ± 84	2598 ± 350
Methyl-THF	AQDS	24 ± 11	bd^a^	5 ± 0	4 ± 2
**Methylene-THF dehydrogenase**					
Methylene-THF	NAD^+^	−^b^	2757304 ±409024	717 ± 19	−^b^
Formaldehyde	NAD^+^	−^b^	bd^a^	bd^a^	−^b^
Methylene-THF	NADP^+^	−^b^	275 ± 35	−^b^	−^b^
Formaldehyde	NADP^+^	−^b^	bd^a^	−^b^	−^b^

*Activities were measured in soluble fraction (SF), membrane fraction (MF) or in SF separated by anion exchange chromatography with a HiTrapQ column (Fraction 1, elution with 200 mM NaCl and Fraction 2, elution with 1 M NaCl). This was done to separate and individually assay the activities of MTHFR and MTHFD. All enzyme assays were performed in triplicates and are given in mU per mg protein. bd^*a*^, below detection limit (<1 mU per mg protein). −^*b*^, not measured.*

MTHFD was measured with methylene-THF and NAD^+^ and NADP^+^ as electron acceptors. Here, the activity with NAD^+^ was 10,000 fold higher than with NADP^+^ and was mainly found in fraction 1. A control experiment with formaldehyde was performed since methylene-THF was synthesized directly in the buffer by addition of THF and formaldehyde. The highest activity here was 5 mU/mg protein in the soluble fraction with NADH.

#### Electron-Carrier Re-oxidizing Enzyme Systems

In an attempt to identify enzyme systems that terminally transfer electrons to protons to release hydrogen or transfer electrons to protons and CO_2_ to release formate, activities of NADH:acceptor oxidoreductase, formate dehydrogenase, and hydrogenase were tested with photometric enzyme assays. An NADH:acceptor oxidoreductase was measured only in the soluble fraction with an activity of 95 mU/mg protein and with the quinone-like artificial electron acceptor AQDS. Activities of formate dehydrogenase and hydrogenase were measured with various electron acceptors (Table 3). Formate dehydrogenase showed very little activity with NAD^+^ (5 mU/mg protein) only in the soluble fraction and not in the membrane fraction. There was no reaction with Fd_ox_. Activity of formate dehydrogenase with AQDS was distributed evenly between soluble fraction (554 mU/mg protein) and membrane fraction (283 mU/mg protein), whereas activity with benzyl viologen was found mainly in the soluble fraction (12234 mU/mg protein in the soluble fraction and 1866 mU/mg protein in the membrane fraction). From these results, formate dehydrogenase can be considered an NAD^+^-independent enzyme. Activity of hydrogenase with NAD^+^ was almost evenly distributed between soluble and membrane fraction. No activity of the hydrogenase was observed with Fd_ox_. When tested with benzyl viologen and AQDS, activities of hydrogenase were found to be enriched in the membrane fraction compared to the soluble fraction.

**TABLE 3:** Activities of formate dehydrogenase and hydrogenase measured with various electron acceptors in the soluble and the membrane fraction of cells grown syntrophically with acetate.

	**Electron carrier**	**SF**	**MF**
Formate DH	AQDS	554 ± 27	283 ± 36
	BV	12234 ± 1161	1866 ± 205
	NAD^+^	5 ± 2	bd^a^
	Fd_ox_	bd^a^	bd^a^
Hydrogenase	AQDS	14 ± 2	254 ± 57
	BV	418 ± 40	1576 ± 116
	NAD^+^	248 ± 34	109 ± 28
	Fd_ox_	bd^a^	bd^a^
NADH:acceptor oxidoreductase	AQDS	95 ± 2	bd^a^

*An NADH: acceptor oxidoreductase was measured with AQDS. Activities were measured in soluble fraction (SF), membrane fraction (MF). All enzyme assays were performed in triplicates and are given in mU per mg protein. bd^*a*^, below detection limit (<1 mU per mg protein).*

## Discussion

In the present study, *T. phaeum* was grown axenically with hydrogen/CO_2_ or formate as well as in a syntrophic co-culture with acetate. All genes of the WLP were found to be expressed during growth with the used substrates. Thus, we confirm that in *T. phaeum* the WLP is used in both directions, depending on the substrate provided (Hattori et al., 2005). In the following part we discuss the enzymes which were prominently induced and thus were putatively connected to energy conservation under the respective growth condition.

### Acetogenic Growth With Hydrogen/CO_2_ or Formate

**FIGURE 5:**
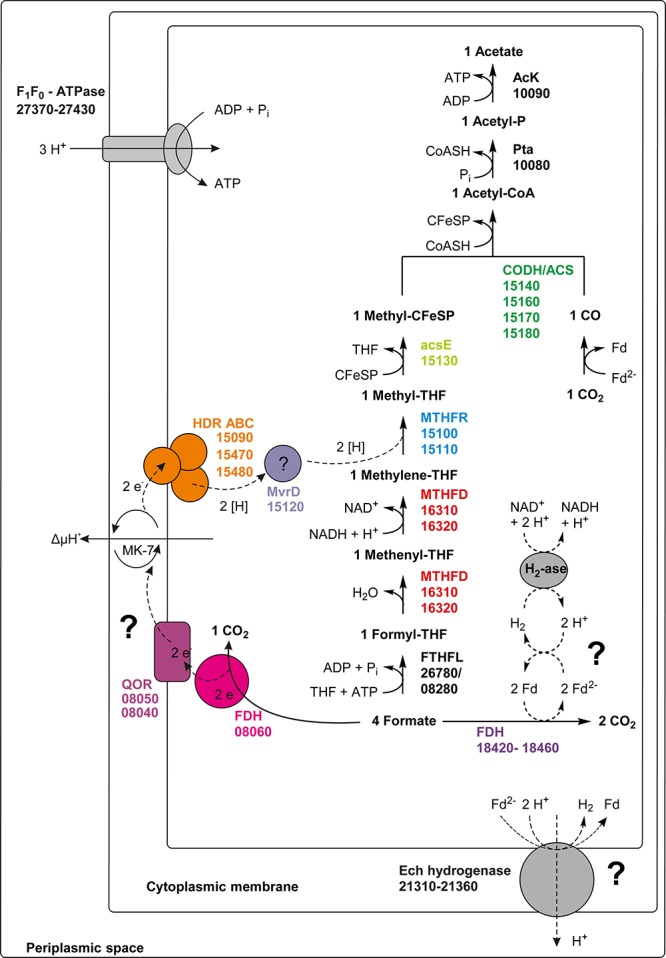
Proposed pathway of formate utilization in *T. phaeum.* Colored squares and circles represent key enzyme systems using the same color coding as in Figure 4. Numbers represent the IMG gene locus tags of proteome-identified enzyme systems. Abbreviations: FeS, iron-sulfur cluster; 4Fe-4S, four-iron-four-sulfur cluster; MoPt, molybdopterin; MK-7, menaquinone-7; Fd, ferredoxin; Fd^2–^, reduced ferredoxin; AcK, acetate kinase; pta, Phosphotransacetylase; CFeSP, corrinoid iron sulfur protein; CODH/ACS, carbon monoxide dehydrogenase/acetyl-coenzyme A synthase-complex; THF, tetrahydrofolate; MTHFR, methylene-THF reductase; MTHFD, methylene-THF dehydrogenase; FTHFL, formyl-THF lyase; HDR, heterodisulfide reductase; FDH, formate dehydrogenase; QOR, quinone:acceptor oxidoreductase; MvrD, methyl-viologen-reducing hydrogenase subunit D; H_2_-ase, hydrogenase. Question marks indicate enzyme systems, whose activities were not detected.

The only acetogen for which the mechanism of energy conservation during growth with hydrogen/CO_2_ was unraveled completely so far is *A. woodii*. In this organism, the energy-conserving enzyme system is the Rnf complex which generates a sodium ion gradient by oxidation of reduced Fd with NAD^+^, thus driving ATP formation (Biegel et al., 2009, 2011; Biegel and Müller, 2010). However, *T. phaeum*, like *M. thermoacetica*, does not contain genes for an Rnf complex in its genome. Another enzyme which was examined as a potential candidate participating in energy conservation is MTHFR. During acetogenesis, the MTHFR reduces methylene-THF to methyl-THF and uses electrons at a potential of −200 mV which can be delivered by NADH [E_0_’(NAD^+^/NADH) = −320 mV] in an exergonic reaction (Schuchmann and Müller, 2014). Indeed, activity of the MTHFR with methylene-THF and NADH was observed in the soluble fraction 2. At first sight, this might appear as evidence that MTHFR is NAD^+^-dependent. Considering the presence of an NADH:AQDS oxidoreductase, this could also mean that NADH is oxidized with quinones or other yet unknown electron acceptors by an NADH:acceptor oxidoreductase (Figure 5). It was suggested that MTHFR could have a bifurcating function and could couple the reduction of Fd_ox_ with NADH to the reduction of methylene-THF. The ΔG_0_’ of the total reaction would be −12 to +2 kJ per mole, depending on the redox potential of the ferredoxin (Köpke et al., 2010; Schuchmann and Müller, 2014). The concept was disproven in *M. thermoacetica* (Mock et al., 2014), and also in the present study such a bifurcating reaction with NADH and Fd_ox_ was not observed. Instead, the observed activity with NADH and methylene-THF can be interpreted as a combined reaction of NADH:acceptor oxidoreductase and MTHFR, i.e., electron transfer from NADH via quinones to methylene-THF. Comparison of MTHFR of *M. thermoacetica* with MTHFR of *T. phaeum* shows high similarity of 55 to 66% (Table 1), but also the whole gene cluster exhibits a similar organization (Figure 4). MTHFR consists of two subunits MetV and MetF whose genes are located next to genes of a hydrogenase. Different from *M. thermoacetica* where all *hdrABC*-genes for the three subunits of the HdrABC complex are located in the same gene cluster, in *T. phaeum* only *hdrA* is located in the *metFV*-gene cluster. Genes *hdrB* and *hdrC* are located next to genes for a putatively periplasmically oriented formate dehydrogenase in a separate gene cluster. Genes *hdrB* and *hdrC* were constitutively expressed during growth with all substrates employed. This indicates that the HdrABC complex functions as a linker between MTHFR and the quinone pool during methylene-THF oxidation and reduction. It was proposed for *M. thermoacetica* that the MTHFR reaction can be coupled to a complex containing Ech hydrogenase plus formate dehydrogenase, similar to the formate hydrogenlyase complex of *E. coli* (Mock et al., 2014). However, no biochemical evidence was provided yet for this concept with *M. thermoacetica* (Mock et al., 2014). During growth with hydrogen/CO_2_, genes coding for a formate hydrogenlyase system were expressed in *T. phaeum* which were not expressed during growth with formate or acetate. It was proposed that the formate hydrogenlyase system of *E. coli* could couple reduction of CO_2_ with hydrogen with the formation of a proton gradient via the membranous HyfBDF subunits (Andrews et al., 1997). Expression of this gene cluster was observed before with *T. phaeum* during growth with ethanol or ethanolamine in axenic cultures (Keller et al., 2019). Under these conditions, CO_2_ reduction via the WLP is used as a sink for electrons derived from ethanol or ethanolamine oxidation to acetate. Under standard conditions, the reduction of CO_2_ to formate with electrons from hydrogen is slightly endergonic which makes it implausible that energy is conserved in this step. During growth with formate, the genes for formate hydrogenlyase system are not expressed, indicating that this system is responsible only for CO_2_-fixation and not for energy conservation. The energy conserving systems during acetogenesis are still unknown. The genes for Ech hydrogenase (Tph_c21310-21360) as a putatively proton-translocating enzyme system are expressed during all growth conditions. Thus this enzyme system is a possible candidate for energy conservation. However, no hydrogenase activity could be measured with Fd_ox_ and hydrogen. A formate dehydrogenase whose gene (Tph_c08060) is located in a gene cluster together with genes of a NADH:quinone oxidoreductase (Tph_c08040- Tph_c08050) was present during growth with formate. The electrons derived in this reaction could be coupled via a quinone pool to the reduction of methylene-THF (Figure 5). However, at least one more formate dehydrogenase needs to be present to deliver low-potential electrons for the CO dehydrogenase. A possible candidate is the constitutively present Tph_c18420.

### Syntrophic Acetate Oxidation

**FIGURE 6:**
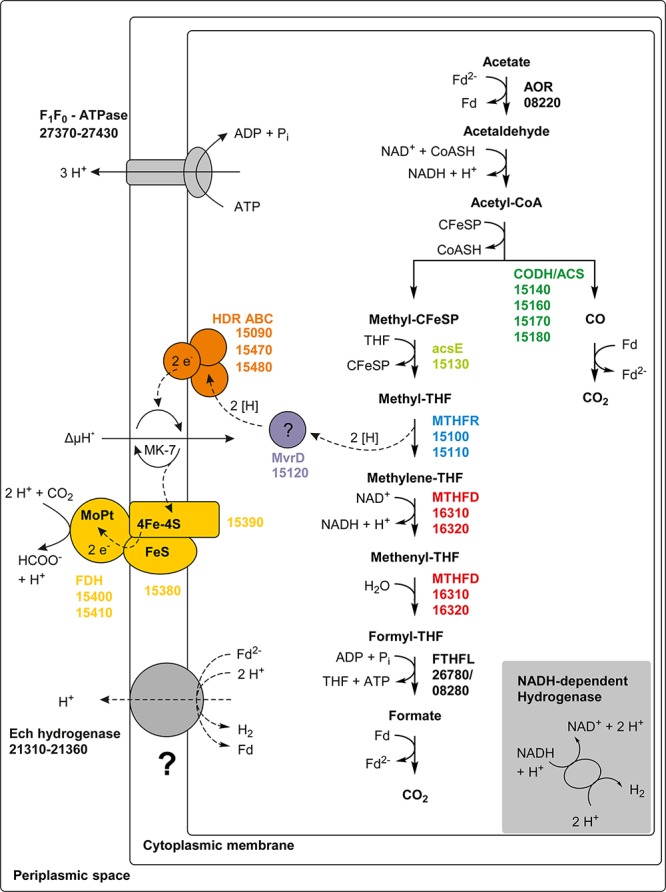
Proposed pathway of acetate oxidation in *T. phaeum.* Colored squares and circles represent key enzyme systems using the same color coding as in Figure 4. Numbers represent the IMG gene locus tags of proteome-identified enzyme systems. Abbreviations: FeS, iron-sulfur cluster; 4Fe-4S, four-iron-four-sulfur cluster; MoPt, molybdopterin; MK-7, menaquinone-7; Fd, ferredoxin; Fd^2–^, reduced ferredoxin; AOR, aldehyde:ferredoxin oxidoreductase; CFeSP, corrinoid iron sulfur protein; CODH/ACS, carbon monoxide dehydrogenase/acetyl-coenzyme A synthase-complex; THF, tetrahydrofolate; MTHFR, methylene-THF reductase; MTHFD, methylene-THF dehydrogenase; FTHFL, formyl-THF lyase; HDR, heterodisulfide reductase; FDH, formate dehydrogenase; MvrD, methyl-viologen-reducing hydrogenase subunit D. Question marks indicate enzyme systems, whose activities were not detected.

In the direction of acetate oxidation, MTHFR poses an energetic barrier as it releases electrons at a redox potential of −200 mV, which cannot be transferred directly to NADH (−320 mV). This thermodynamic situation is comparable to ethanol oxidation with NAD^+^ by alcohol dehydrogenase and is thus hardly possible at high product concentrations (Schmidt et al., 2014). However, the MTHFR in *T. phaeum* appears to be NAD^+^-independent, at least in the direction of methyl-THF oxidation. For growth of *T. phaeum* with hydrogen/CO_2_, it was suggested that MTHFR could be linked to a quinone pool by an HdrABC system. The HdrB and HdrC subunits are encoded in a gene cluster together with genes for a periplasmically oriented formate dehydrogenase. This formate dehydrogenase is present only during growth with acetate, and it reveals high similarity to a previously described periplasmically oriented formate dehydrogenase in *S. wolfei* (Schmidt et al., 2013; Crable et al., 2016). In *S. wolfei*, this gene cluster is expressed during syntrophic butyrate oxidation. The electrons from butyryl-CoA oxidation to crotonyl–CoA have a comparably high electron potential of E_o_’ = −10 mV or −125 mV depending on the literature (Gustafson et al., 1986; Sato et al., 1999) and thus cannot be used directly for NAD^+^ reduction. In *S. wolfei*, an electron transfer flavoprotein (EtfAB) carries the electrons from butyryl-CoA dehydrogenase to a membrane-bound FeS-containing oxidoreductase which reduces the quinone pool in the membrane (Schmidt et al., 2013; Crable et al., 2016). In *T. phaeum*, the MTHFR activity with BV and AQDS as artificial electron acceptors was found in washed membrane fractions indicating that MTHFR is associated with the membrane, yet not membrane-integral as it lacks transmembrane helices. Probably the enzyme whose gene is annotated as coenzyme F_420_-reducing hydrogenase (Tph_c15120) transfers the electrons to the HdrABC system which subsequently reduces a quinone, most likely menaquinone, as menaquinone MK-7 is the predominant quinone in *T. phaeum* (Hattori et al., 2000; Oehler et al., 2012). This system thus produces methylene-THF and menaquinol. The latter could then be re-oxidized at the gamma subunit of the formate dehydrogenase. The electrons are transferred to an iron-sulfur cluster-containing subunit and finally to the active site of the formate dehydrogenase (Figure 6). We show here that *in vitro* activity of a quinone-dependent formate dehydrogenase can be measured with BV or with AQDS as an artificial quinone. Similar enzyme assay results were obtained for hydrogenase, which is apparently also NAD^+^-independent, quinone-dependent and membrane bound. One potential candidate could be a complex of three hydrogenase subunits, which were constitutively present in the proteome (Figure 2, Tph_c06350 – Tph_c06370). One subunit (Tph_c06350) carries a TAT-signal sequence and another subunit (Tph_c06370) has transmembrane helices and is annotated as b-type cytochrome subunit that is most likely responsible for redox communication with menaquinone. This protein complex hence possibly resembles a membrane-bound, periplasmically oriented and quinone-dependent hydrogenase analogous to the described formate dehydrogenase. Therefore, besides formate, electrons derived from methyl-THF oxidation could alternatively be released as hydrogen via menaquinone similar to the system in *S. wolfei* (Crable et al., 2016). Yet, proteome data of *T. phaeum* obtained in the current study indicates, that membrane-bound formate dehydrogenase is of greater importance, as it is the only enzyme system that is almost exclusively present during syntrophic growth with acetate (Figure 2). Coupling MTHFR to the putatively periplasmically oriented formate dehydrogenase could overcome the energetic barrier that this reaction sets in the reversed WLP. However, this reaction would need to be pulled by a proton gradient and a low formate concentration. The low formate concentration can be achieved only in syntrophic cooperation with *M. thermautotrophicus* strain TM as partner that uses both formate and hydrogen as electron donors (Hattori et al., 2001). This could explain why *T. phaeum* has difficulties to oxidize acetate with a methanogen that uses only hydrogen as electron donor (Hattori et al., 2001). Unfortunately, formation of a proton gradient coupled to methyl-THF oxidation by quenching of the fluorescent dye ACMA in inverted membrane vesicles according to Schoelmerich and Müller (2019) could not yet be demonstrated in *T. phaeum* (data not shown). The postulated reversed electron transport from methyl-THF to formate would require a proton gradient to be established by ATP hydrolysis. This would mean that acetate cannot be activated to acetyl-phosphate with acetate kinase as typical of the acetate-forming WLP (Schuchmann and Müller, 2014). Alternatively, acetate could be activated by an acetaldehyde oxidoreductase without ATP investment as it was described before for *Clostridium ljungdahlii* (Köpke et al., 2010; Bengelsdorf et al., 2013; Keller et al., 2019). The ATP thus “saved” could be partly invested into the described reversed electron transport system. In cell-free extracts of *T. phaeum*, the activity of acetaldehyde oxidoreductase was proven with benzyl viologen as electron acceptor in the direction of acetaldehyde oxidation (Keller et al., 2019). In the physiological direction of acetate reduction, no activity could be measured yet. A reason for this failure could be the presumably low activity of the enzyme in the direction of acetaldehyde formation. Experiments with a purified aldehyde:ferredoxin oxidoreductase (AOR) of *M. thermoacetica* indicate that acetate (K_m_ = 5.6 mM) is turned over at an about 500 times higher K_m_ than acetaldehyde (K_m_ = 10 μM) (Huber et al., 1995). With this, an accumulation of toxic acetaldehyde inside the cell is avoided. Attempts were made to purify the acetaldehyde:oxidoreductase of *T. phaeum*, however, no active protein fraction was obtained so far (data not shown).

Recently, a genomic comparison of the five AOB sequenced to that date, i.e., *C. ultunense* (Schnürer et al., 1996), *T. phaeum* (Hattori et al., 2000), *Pseudothermotoga lettingae* (Balk et al., 2002), *S. schinkii* (Westerholm et al., 2010) and *Tepidanaerobacter acetatoxydans* (Westerholm et al., 2011) was published (Manzoor et al., 2018). This study revealed that not all SAOBs use the WLP. Even *P. lettingae* and *C. ultunense* lack central enzymes of the WLP such as the CODH/ACS and MTHFR in their genomes, thus more than one pathway of acetate oxidation must exist (Manzoor et al., 2018). Only *S. schinkii* and *T. phaeum* encode the entire WLP, but *S. schinkii* is not able to grow with hydrogen/CO_2_ or formate. According to Manzoor et al. (2018), *T. phaeum* is the only SAOB that encodes a formate hydrogenlyase system and only *S. schinkii* encodes also a membrane-bound formate dehydrogenase (Ssch_1490003-1490006). The periplasmically oriented formate dehydrogenase and the formate hydrogenlyase complex, both representing two membrane-bound enzyme systems, could be the key for the reversibility of the WLP in *T. phaeum*. However, the exact mechanism of these systems is unclear, and it is indispensable to provide further biochemical data additional to genomic and proteomic studies to support the proposed fermentation pathways.

## Data Availability Statement

The raw data supporting the conclusion of this manuscript will be made available by the authors, without undue reservation, to any qualified researcher.

## Author Contributions

AK conducted the experiments designed by AK and NM. AK, NM, and BS wrote and approved the final manuscript.

## Conflict of Interest

The authors declare that the research was conducted in the absence of any commercial or financial relationships that could be construed as a potential conflict of interest.

## Footnotes

1 https://img.jgi.doe.gov/cgi-bin/m/main.cgi

2 http://www.cbs.dtu.dk/services/TMHMM/

3 http://www.cbs.dtu.dk/services/SignalP/

4 http://genomics.unl.edu/bSECISearch

## Supplementary Material

TABLE S1 Total Proteomics analysis of cells of *Thermacetogenium phaeum* grown with formate (Formiat), hydrogen/CO_2_ (H_2_CO_2_), or acetate (Acetat). Shown are the results of mass spectrometry analysis using the Proteome Discoverer software (Thermo Fisher). Identified proteins are presented along with the accession numbers of their genes as well as the MASCOT-scores and the area values of the Proteome Discoverer software (Thermo Fisher). Area values were used for semi-quantitatively presenting the abundances of the identified proteins. The data for cells grown with acetate are the same as used in (Keller et al., 2019).
